# The Feasibility of Societal Cost Equivalence between Robotic Hysterectomy and Alternate Hysterectomy Methods for Endometrial Cancer

**DOI:** 10.1155/2011/570464

**Published:** 2011-11-15

**Authors:** Neel T. Shah, Kelly N. Wright, Gudrun M. Jonsdottir, Selena Jorgensen, Jon I. Einarsson, Michael G. Muto

**Affiliations:** ^1^Division of Minimally Invasive Gynecologic Surgery, Department of Obstetrics and Gynecology, Brigham and Women's Hospital, Boston, MA 02115, USA; ^2^Harvard Medical School, Boston, MA 02115, USA; ^3^Division of Gynecologic Oncology, Department of Obstetrics and Gynecology, Brigham and Women's Hospital, Boston, MA 02115, USA

## Abstract

*Objectives*. We assess whether it is feasible for robotic hysterectomy for endometrial cancer to be less expensive to society than traditional laparoscopic hysterectomy or abdominal hysterectomy.* Methods*. We performed a retrospective cohort analysis of patient characteristics, operative times, complications, and hospital charges from all (*n* = 234) endometrial cancer patients who underwent hysterectomy in 2009 at our hospital. Per patient costs of each hysterectomy method were examined from the societal perspective. Sensitivity analysis and Monte Carlo simulation were performed using a cost-minimization model. *Results*. 40 (17.1%) of hysterectomies for endometrial cancer were robotic, 91 (38.9%), were abdominal, and 103 (44.0%) were laparoscopic. 96.3% of the variation in operative cost between patients was predicted by operative time (*R* = 0.963, *P* < 0.01). Mean operative time for robotic hysterectomy was significantly longer than other methods (*P* < 0.01). Abdominal hysterectomy was consistently the most expensive while the traditional laparoscopic approach was consistently least expensive. The threshold in operative time that makes robotic hysterectomy cost equivalent to the abdominal approach is within the range of our experience. *Conclusion*. It is feasible for robotic hysterectomy to be less expensive than abdominal hysterectomy, but unlikely for robotic hysterectomy to be less expensive than traditional laparoscopy.

## 1. Introduction

The burden of endometrial cancer worldwide is significant, particularly in developed nations battling concomitant epidemics of obesity [1]. In the USA and Europe, endometrial cancer has become the most common gynecologic malignancy and the fourth most common cancer site overall [2]. The primary treatment for endometrial cancer is total hysterectomy, bilateral salpingoopherectomy, and surgical staging [3, 4]. In the USA, the total annual cost of this surgery approximates $250 million [5].

Until the advent of operative laparoscopy, the traditional approach to the surgical management of endometrial cancer was total abdominal hysterectomy (TAH). With the diffusion of laparoscopic technology, there has been an increasing trend towards minimally invasive methods [6]. Several large randomized trials have demonstrated equivalent safety and short-term clinical outcomes between TAH and laparoscopic hysterectomy for endometrial cancer, including the Gynecologic Oncology Group's LAP-2 trial and the Australian LACE trial [7, 8]. Furthermore, these trials have demonstrated favorable quality of life outcomes for laparoscopic hysterectomy compared to TAH in terms of shorter hospital stays, less pain, and faster resumption of daily activities.

As a result of shorter hospitalization, for many patients, total laparoscopic hysterectomy (TLH) is likely to be more cost effective to society than TAH in terms of incremental costs per complication-free patient—however, this may not be the case in obese patients with BMI > 35 due to high laparotomy conversion rates [9, 10]. Robot assisted total laparoscopic hysterectomy (TRH) is an increasingly available alternative that may confer an advantage in obese and other complex patients by lowering the need for conversion. Successful TRH for endometrial cancer in the setting of extreme obesity (BMI 98) has been described in the literature with patient discharge on postoperative day one [11]. Moreover, several retrospective studies have demonstrated equivalent safety and short-term clinical outcomes between TRH and alternate methods [12–15].

Despite evidence demonstrating equivalent safety and efficacy between TAH, TLH, and TRH for correctly selected patients, it remains uncertain how the robotic approach compares to other methods in terms of societal costs. Robotic surgery costs were found to be significantly higher than traditional laparoscopy for staging of endometrial cancer in a small cohort of patients [16]. A modeling study based on literature-review-derived parameter estimates verified this finding, but also found that TRH is less expensive than TAH when recovery time is taken account from the societal perspective [17]. In this paper, we use a large cohort from our institution to directly model the feasibility of cost equivalence between TRH and alternate hysterectomy methods from the societal perspective.

## 2. Materials and Methods

### 2.1. Data Collection

All hysterectomies performed for endometrial cancer at the Brigham and Women's Hospital from January 1, 2009 to December 31, 2009 were obtained from operating room (OR) case records. Only cases performed by our four subspecialty-trained gynecologic oncologists were included. 234 women were identified who underwent TAH, TLH, or TRH for endometrial cancer (vaginal hysterectomy is not performed in patients with preoperative diagnosis of endometrial cancer at our institution as this mode of access precludes cytology and further staging). The Brigham and Women's Hospital is an academic tertiary care center in Boston, Mass, USA. Our operating room is divided into pods by specialty, so all supervising anesthesiologists, circulating nurses, and scrub technicians were familiar with gynecologic procedures. With the exception of four cases, all surgeries included both residents and fellows as surgical assistants. All patients were cared for postoperatively by a single group of nurses on a gynecology women-only unit in our institution.

With Institutional Review Board (IRB) approval from the Brigham and Women's Hospital, information was abstracted from electronic medical records, and reviewed for each patient. The information obtained from the charts included the medical record number, date of birth, age, insurance carrier, body mass index (BMI), parity, preoperative and postoperative diagnoses, procedure performed, pathology report, length of stay, surgeon, assistant type (attending, fellow, or resident), estimated blood loss (EBL), indication for surgery, prior abdominal surgery, uterine weight, postoperative complications, postoperative admission, intraoperative complications, conversions, and “OR time.” It is important to note that “OR time” was defined as the total in-room to out-of-room time instead of the commonly reported “skin-to-skin” surgical time because the cost of utilizing an OR is based on the total time the room is occupied. Therefore, in addition, the length of time taken to perform the surgery, “OR time,” includes time taken to intubate, position, and otherwise prepare prior to skin incision, as well as the time of patient recovery in the OR.

Intraoperative complications were defined as bowel, urological, or vascular injuries, or EBL > 1000 mL [18]. Hospital accounting ledgers were used to obtain operative and total encounter billing charges. Statistical descriptions and analyses were performed using SAS software (SAS Institute Inc., Cary, NC, USA; Version 9.0). Associations were tested using *χ* ^2^ and Fisher exact tests for categorical variables, and with analysis of variance and two-sided Student's *t*-tests with Bonferroni correction for continuous variables where appropriate. A multivariate linear and logistic regression analysis was also performed for the variables under study as they relate to the outcomes and costs as listed. A *P* value <0.01 was considered significant for all variables.

### 2.2. Cost Approximation

All costs were approximated from the societal perspective. Using return to work following surgery as the endpoint, societal costs include the direct amount paid for surgery and associated hospitalization as well as indirect lost labor production costs (foregone wages from work absence while patients were recovering). The amount that is reimbursed for a set of health services varies considerably in the USA depending on a multitude of individually negotiated agreements between public and private payer organizations. Although the amount paid is usually less than the standard hospital charges, hospital charges provide an upper-bound estimate. In our cohort, 63 (26.9%) patients had health insurance through Medicare or Medicaid with well-defined reimbursement rates, 2 (0.9%) patients were self-pay responsible for the full charge, and the remaining 169 (72.2%) had private insurance with varying negotiated reimbursements.

Hospital charges therefore overestimate the absolute average amount paid. However, because our goal is to determine whether it is at least feasible for TRH to be less expensive than alternate hysterectomy methods, we use hospital charges as a reliable upper-bound estimate because it preserves relative cost differences between hysterectomy methods in the most expensive scenario. Moreover, since hospital charges are determined in an identical manner for each patient in our cohort, using hospital charges (rather than individually negotiated reimbursements) to approximate payments maintains the internal validity of our analysis.

The amount charged by our hospital for surgery was obtained from hospital accounting ledgers and was determined based on direct costs (equipment cost and OR time) and indirect costs in 5 categories (day surgery bed occupancy, ambulatory procedure room occupancy if applicable, recovery room bed occupancy, nursing staff, and nonnursing staff). Cost based on OR time (total time use of the operating room from when the patient enters the OR to when the patient leaves) is calculated according to a graduated scale fee schedule that bills in 15-minute increments. This fee schedule is based on a combination of labor, supplies, and fixed equipment for an operating room and is adjusted every calendar year. Equipment cost is calculated based upon standardized sets of equipment and supplies commonly used by high-volume surgeons at our institution called “case carts.” Any surgeon can request a standardized hysterectomy “case cart” and subsequently add the cost of his or her desired disposable tools. The cost of what the hospital pays for all disposables and the depreciation cost of nondisposables are itemized for each case. The cost of the equipment is then multiplied by a graduated fee to create a patient charge. The cost of robotic equipment is similarly amortized based on depreciation cost and accounted for with a flat fee for every robot case.

Nonoperative charges for the associated hospitalization are based on direct (labor, variable supplies including pharmacy, laboratory, and blood bank, fixed equipment) and indirect costs (room and board based on cost of depreciation per square foot of unit) related to the encounter. Surgeon and anesthesia professional fees are based on the appropriate Current Procedural Terminology (CPT) codes for the appropriate procedures (e.g., surgeon codes 58150 for TAH, or 58570 for TLH and TRH, etc.).

The additional indirect societal costs for each patient were approximated as the cost of work “absenteeism” [17]. Average foregone wages from work absence, using median wages reported by the National Bureau of Labor Statistics and the published average return to work for patients who undergo each hysterectomy method were calculated. Work absences were valued using the median gender-specific weekly salary for full-time workers ages 19 years and older, accounting for the national unemployment rate in 2009. Median weekly earnings were $657. Average USA work absence per method of hysterectomy was obtained. Previously published mean return to work times are 38–41 days after TAH [19, 20]. Return to work after TLH hysterectomy is 19–24 days according to both our institutional data and previously published data [21, 22]. Limited data exist approximating return to work after TRH, though one study suggests that return to normal activity may be faster for TRH compared to TLH in a small cohort of patients [23]. Given limited data and that no empirical reason to assume TRH return to work would be faster than TLH in our cohort based on number and size of incisions, we assumed equivalent return to work times between TRH and TLH (19–24 days) [21, 22].

### 2.3. Cost Analysis

**Figure 1:**
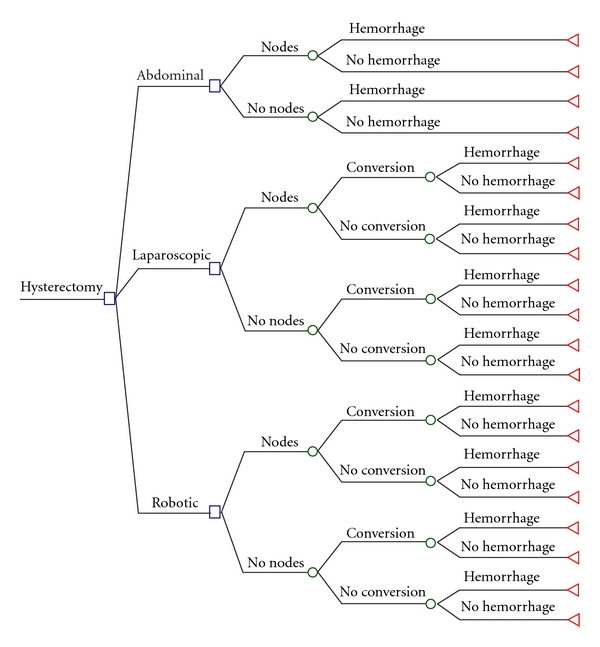
Cost analysis decision tree. Decision model tree structure showing comparison of abdominal (TAH), laparoscopic (TLH), and robotic (TRH) hysterectomy methods. The squares denote decision nodes, circles denote probability nodes, and triangles denote the terminal nodes. All probabilities and operating room times are based on our clinical experience in 2009 as summarized in Tables 1 and 2. The outcome “nodes” in the model refer to lymph node dissection, and “hemorrhage” refers to EBL > 1000 mL.

To compare the costs of TAH, TLH, and TRH, we created a decision tree model, as described by the cost-analysis guidelines of the National Information Center on Health Services Research and Health care Technology (NICHSR) (Figure 1) [24]. Given the assumption of equivalent short-term “effect” (i.e., successful removal of the uterus) by each method, we conducted a cost-minimization analysis rather than a cost-effectiveness analysis. The cost analysis was performed from the perspective of societal costs as outlined above. Relevant clinical outcomes included in the decision tree (need to convert to laparotomy and estimated blood loss > 1000) were chosen based on a previously published endometrial cancer hysterectomy cost decision tree model [17]. Moreover, this model is also consistent with a previously established institutional cost model at our hospital, which found that the overwhelming majority of variation in operative cost was driven by OR time and that the primary time-extending variables were the need to convert to laparotomy and intraoperative complication rate [25]. We added the decision to perform lymph node dissection as an additional variable for two reasons: (1) at our institution lymph node, dissection is not performed universally for all cases and (2) our institution bases OR charges on 15-minute increments as detailed above, and lymph node dissection added greater than 15 minutes to the OR time in our experience. Based on the finding that operative costs are primarily driven by OR time, our cost model makes the additional assumption that the added cost of doing a lymph node dissection, needing to convert to laparotomy, or having an intraoperative complication such as hemorrhage is equivalent to the cost of extended operative time required.

All decision tree analysis was performed using TreeAge Software (TreeAge Inc., Williamstown, Mass, USA). The comparative costs of TAH, TLH, and TRH were calculated by multiplying the costs associated with each clinical outcome by the probabilities of the occurrence of that outcome and then adding the nonoperative hospital charges and expected indirect societal costs as outlined above. Probabilities and costs were directly applied from our cohort database and hospital accounting ledgers. Our model was tested deterministically using one-way sensitivity analyses to assess the effects of varying OR time over the ranges experienced by our cohorts, as well as probabilistically using Monte Carlo simulation (Figure 3). To assess the uncertainty of our estimates using Monte Carlo simulation, normal probability distribution functions were substituted for each clinical outcome variable in the decision tree based on cohort mean and standard deviation statistics. Given no robotic conversions in our cohort, previously reported summary statistics were used to create probability distribution functions for Monte Carlo simulation in this case [17].

## 3. Results

The characteristics of patients who underwent hysterectomy by each method are summarized in Table 1. The average age across all three cohorts was similar. Those who underwent TRH tended to have the highest BMI (40.5, *P* < 0.01), while those who underwent TLH tended to have lowest BMI (29.8, *P* < 0.01). On average, patients who underwent TLH were less surgically complex than patients who underwent hysterectomy by other methods. These patients tended to have the lowest incidence of prior laparotomy, prior laparoscopy, and presence of adhesions. Moreover, patients selected for TLH had the lowest average uterine weight (134.4 g). By contrast, patients selected for TAH had the greatest average uterine weight (243.5 g). Patients who underwent TAH were also significantly more likely to also undergo staging with lymph node dissection (74%, *P* < 0.01), compared to TLH (38.5%) and TRH (37.9%). Multivariate regression demonstrated a small correlation between lymph node dissection and hysterectomy type (*R* = 0.3, *P* < 0.01), as well as uterine weight and hysterectomy type (*R* = 0.18, *P* < 0.01).

Perioperative outcomes and complications are summarized by hysterectomy type in Table 2. On average, TRH required the longest use of the operating room (including the skin-to-skin surgical time, setup, and turnover times), totaling 252.6 minutes (*P* < 0.01). Estimated blood loss was significantly lower for patients who underwent TLH and TRH compared to patients who underwent TAH (*P* < 0.01). Compared to patients who underwent TAH, patients who underwent TLH and TRH also stayed in the hospital for a significantly lower amount of time (*P* < 0.01), consistent with length of stays reported elsewhere in the literature [12, 15, 26].

The rate of conversion to laparotomy was 5.1% for TLH and 0% for TRH. Intraoperative complication rate was very low with single incident of organ injury for TAH and TLH (bladder injury in both cases) and a single incident of EBL greater than 1000 mL reported for TAH and TLH, and no complications reported for TRH. Postoperative complication rates were similar across all three cohorts.

Cost approximations are summarized in Table 3, based both on hospital accounting ledgers for each patient in our database (total mean operative charge and total mean encounter charge), as well as our cost model which additionally accounts for variation in OR time and indirect societal costs (expected societal cost). As described in Section 2, these cost approximations are upper-bound estimates based on standard hospital charges. Notably, analysis of variance demonstrated that operative charges were most strongly driven by operating room time (*P* < 0.01) with an *R*-value of 0.963. No other variable was significantly associated with operative charges, and the charges associated with time use were 190-fold greater than charges associated with equipment. It is therefore not surprising that the total mean operative charge for TRH is the highest (*P* < 0.01), given that TRH also required the longest time use of the operating room. 

Mean encounter charge is highest for TAH (*P* < 0.01) as a result of longer lengths of stay in the hospital. The expense of TAH increases relative to the minimally invasive methods when return to work times are taken into account, and foregone wages are added to the indirect societal costs of the procedure. Ultimately, the expected total societal costs are highest for TAH and lowest for TLH. 

**Figure 2:**
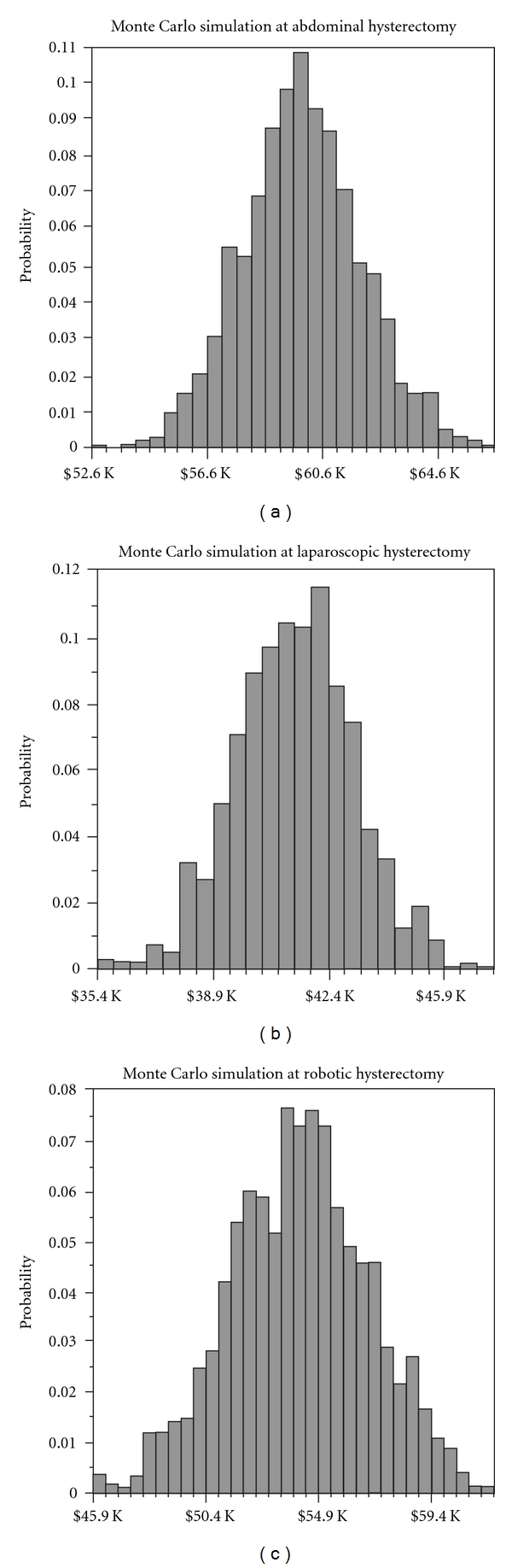
Monte Carlo simulation of total expected societal costs. Expected total societal costs as determined by our model for each hysterectomy method are reported in Table 3. Below are three corresponding Monte Carlo simulations demonstrating the expected probability distribution of total societal costs, with total societal costs on *x*-axis and probability on *y*-axis.

Each probability and outcome variable in our decision tree were assigned normally distributed probability functions and then randomly sampled in a probabilistic Monte Carlo simulation in order to test the robustness of our expected societal cost estimate.  Figure 2 demonstrates the distribution of societal costs estimates for each method of hysterectomy. In all cases, the simulation resulted in estimates that were closely clustered around our expected values. 

**Figure 3:**
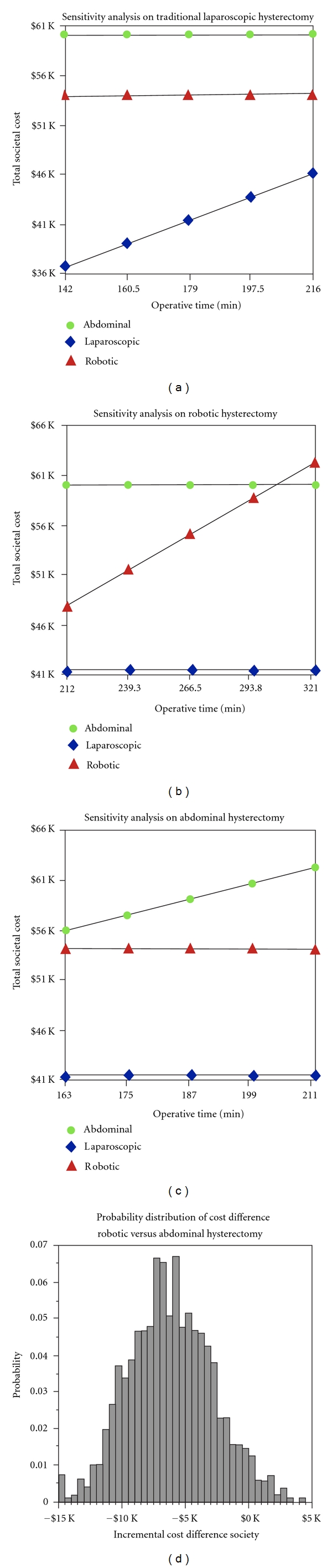
Sensitivity analysis on operating room time. (a)–(c) demonstrate one-way sensitivity analysis on operating room time, ranging from the 25th to 75th percentile of our experience. Operating room time is on the *x*-axis, and total societal cost is on the *y*-axis. TAH is labeled with green circles, TLH is labeled with blue diamonds, and TRH is labeled with red triangles. (d) is a Monte Carlo simulation demonstrating the incremental cost difference to society on the *x*-axis of TRH compared to TAH (negative cost means that TRH is less expensive than TAH, while positive cost means that TRH is more expensive than TAH), and probability of that outcome on the *y*-axis.

We then individually conducted a one-way sensitivity analysis on operating room time for each method of hysterectomy. In each case, as operative room time was allowed to increase, the expected total societal cost increased as well.  Figure 3(a) demonstrates that, even for longer cases in our cohort, the expected societal cost of TLH is less than the expected costs of TRH or TAH.  Figure 3(b) demonstrates that, over the majority of our experience in operative time, TRH is less expensive than the average expected societal cost of TAH, but still more expensive than TLH. Moreover, TRH becomes more expensive than TAH when the operating room is utilized for more than approximately 300 minutes (including the time of anesthesia induction and intubation, equipment setup, etc.) Figure 3(c) demonstrates that the fastest TAH cases in our experience approach the average expected societal cost of TRH and are more expensive than TLH by a wide margin.  Figure 3(d) shows a Monte Carlo simulation of the expected incremental cost difference to society between TRH and TAH. In the majority of cases, the estimated societal cost of TRH is less than the estimated societal cost of TAH. 

In all cases, TLH was consistently the least costly method to society. In our experience, it is feasible for TRH to be less expensive to society than TAH, but unlikely for TRH to be less costly than TLH. 

## 4. Discussion

There are many reasons why a surgeon may prefer a particular method of hysterectomy for a given patient with endometrial cancer. Conversely, there are many patients for whom TAH, TLH, and TRH might all be appropriate. Particularly in patients for whom more than one method would be equivalently safe and effective, it has become increasingly important for healthcare providers to take societal cost into account [27]. 

Elucidating the costs of procedures in the American healthcare system is seldom straightforward given the discordance between the cost of providing a service, the amount a patient is charged, and the amount a provider is reimbursed. For that reason, the aim of our paper was not to determine the absolute societal costs of hysterectomy for endometrial cancer, but to assess the relative cost differences between hysterectomy methods. In particular, we sought to determine whether it is at least feasible for TRH to be less expensive to society than TLH or TAH. 

To preserve consistency in the expected amount paid for each patient, we used hospital charges as an upper-bound estimate of our cost approximations. Other papers have converted hospital charges to hospital costs using a multiplier of 0.5–0.7 as the “cost-to-charge ratio” [17, 28, 29]. Although the absolute costs we describe here are higher than those reported elsewhere in the literature, if we had applied this adjustment, our expected societal costs would be similar to those previously published [5]. We did not feel that it was necessary to apply this adjustment however as the cost to charge relationship is often arbitrary and subject to widespread regional variation within the USA. Moreover, by using charges to provide upper-bound societal cost estimates, we demonstrate that it is feasible for TRH to be less costly to society than TAH even in the most expensive payment scenario. 

The OR utilization times we report in our study (including setup, surgical time, and immediate recovery time) may be longer than those experienced at institutions without similar teaching missions. With rare exception, almost all patients had both resident and fellow involvement as assistants in their surgery. In addition, TRH is performed selectively at our institution and is primarily reserved for high-BMI patients, where longer operative times are expected compared to patients with lower BMI. 

Our institutional experience-based model offers several advantages. First, it likely reflects the experience of other academic tertiary care centers that assess charges in a similar manner. Second, while the other previously published cost-minimization model essentially doubles the cost of cases that are converted to laparotomy, we were able to assess the cost of conversion and intraoperative complications more directly by using the cost of extended time required from our experience [17]. 

A primary limitation of our study design is that we examine our cohorts retrospectively. As a result, there is an inherent selection bias as patients appear to be selected for certain hysterectomy methods based on surgical complexity. For instance, patients selected for TAH are more likely to have advanced disease requiring lymph node dissection and were also more likely to have higher uterine weight and adhesions from prior surgery. Ideally, the relative merits of each method (including the expected societal costs) will be assessed with a randomized control trial. To date, no such trial exists. 

The margin of benefit to performing TRH in terms of cost efficiency is likely to improve as surgeons become increasingly familiar with this technology. Our data demonstrates that our fastest TRH cases are substantially less expensive than TAH. Since cost accrues for total OR time utilization, there may also be opportunities to improve the efficiency of setting up, docking, and disassembling the robot. 

Our study demonstrates that it at least feasible for TRH to be less expensive than TAH. We also find that TLH is consistently the least costly method to society, a finding supported by the previously published literature [17, 23]. Our institutional experience therefore suggests that it may be best to perform TLH when possible to optimize societal cost savings. However, if TLH is precluded by extreme obesity or some other complexity, TRH is preferable from the societal perspective to TAH.

## Figures and Tables

**Table 1:** Cohort characteristics. Number and % or mean and standard deviation (range).

Characteristics	TAH *n* = 73 (31.2%)	TLH *n* = 118 (50.4%)	TRH *n* = 43 (18.4%)
Age (years)	61.9, 9.2 (43–87)	59.9, 10.4 (34–91)	58.2, 7.57 (43–74)
BMI (kg/m^2^)	35.7, 10.1 (16.7–69.4)	29.8*, 7.5 (19.7–59.2)	40.5*, 11.0 (18.6–61.4)
Prior laparotomy	32 (46.4%)	35 (31.5%)	19 (46.3%)
Prior laparoscopy	13 (19.7%)	11 (10.5%)	10 (23.8%)
Adhesions	24 (32.9%)	17 (14.4%)	17 (39.5%)
Uterine weight (g)	243.5, 330.12 (25–2170)	134.4, 101.4 (34–704)	176.3, 153.34 (45.5–905)
Lymph Node Dissection (LND)	54 (74.0%)*	45 (38.5%)	16 (37.9%)

TAH: total abdominal hysterectomy, TLH: total laparoscopic hysterectomy, and TRH: total robotic hysterectomy; standard deviation reported where % does not occur. *P < 0.01.

**Table 2:** Perioperative outcomes and complications by hysterectomy type. Mean (SD) and range or number and (%).

	TAH	TLH	TRH
OR time (min)	192.28 (181.08, 203.48)	186.80 (177.997, 195.61)	252.6^∗‡^(238.01, 267.19)
Estimated blood Loss (mL)	255.94 (215.74, 296.14)	105.23* (73.30, 137.07)	41.22* (−13.68, 96.12)
Length of Stay (days)	3.84 (3.46, 4.21)	1.44* (1.16, 1.72)	1.30* (0.83-1.77)
Conversions	—	6 (5.1%)	0
Organ injury	1 (1.37%)	1 (0.85%)	0
EBL ≥ 1000	1 (1.45%)	1 (0.91%)	0
Postoperative complications^†^	8 (11.0%)	8 (6.8%)	3 (7.0%)

TAH: total abdominal hysterectomy, TLH: total laparoscopic hysterectomy, and TRH: total robotic hysterectomy.

*P < 0.001 using one-way analysis of variance (TLH and TRH compared to TAH).

^‡^ *P* < 0.001 using one-way analysis of variance (TRH compared to TLH).

^†^Major (readmission, reoperation, ileus, hemorrhage) and minor complications (infections; chest, urinary, wound).

**Table 3:** Total cost estimates.

	TAH	TLH	TRH
Mean operative charge	$33,756	$33,706	$44,698*
Mean encounter charge	$54,110*	$39,367	$51,552*
Expected societal cost	$59,997	$41,339	$54,062

TAH: total abdominal hysterectomy, TLH: total laparoscopic hysterectomy, and TRH: total robotic hysterectomy; *P < 0.01. Uncertainty in the expected societal costs is assessed using a Monte Carlo simulation (Figure 2).
